# The Different Sensitive Behaviors of a Hydrogen-Bond Acidic Polymer-Coated SAW Sensor for Chemical Warfare Agents and Their Simulants

**DOI:** 10.3390/s150818302

**Published:** 2015-07-28

**Authors:** Yin Long, Yang Wang, Xiaosong Du, Luhua Cheng, Penglin Wu, Yadong Jiang

**Affiliations:** State Key Laboratory of Electronic Thin Films and Integrated Devices, School of Optoelectronic Information, University of Electronic Science and Technology of China (UESTC), Chengdu 610054, China; E-Mails: yinlong0506@foxmail.com (Y.L.); uestcwang@126.com (Y.W.); cheng.luhua@foxmail.com (L.C.); uestcstuwpl@163.com (P.W.); jiangyd@uestc.edu.cn (Y.J.)

**Keywords:** hydrogen-bond acidic polymer, chemical sensor, SAW

## Abstract

A linear hydrogen-bond acidic (HBA) linear functionalized polymer (PLF), was deposited onto a bare surface acoustic wave (SAW) device to fabricate a chemical sensor. Real-time responses of the sensor to a series of compounds including sarin (GB), dimethyl methylphosphonate (DMMP), mustard gas (HD), chloroethyl ethyl sulphide (2-CEES), 1,5-dichloropentane (DCP) and some organic solvents were studied. The results show that the sensor is highly sensitive to GB and DMMP, and has low sensitivity to HD and DCP, as expected. However, the sensor possesses an unexpected high sensitivity toward 2-CEES. This good sensing performance can’t be solely or mainly attributed to the dipole-dipole interaction since the sensor is not sensitive to some high polarity solvents. We believe the lone pair electrons around the sulphur atom of 2-CEES provide an electron-rich site, which facilitates the formation of hydrogen bonding between PLF and 2-CEES. On the contrary, the electron cloud on the sulphur atom of the HD molecule is offset or depleted by its two neighbouring strong electron-withdrawing groups, hence, hydrogen bonding can hardly be formed.

## 1. Introduction

Trace level detections of harmful or toxic chemicals, such as nerve agents [1,2,3,4,5,6,7,8,9,10,11], explosives [12,13,14,15,16,17,18], and volatile organic compounds (VOCs) [19,20,21,22,23], have been intensely studied by means of a wide variety of sensing technologies. In general, application of chemical sensors in this area requires coating of a sorptive material onto the surface of specific transducers [1,2,3,4,5,6,7,8,16,17,18,19,20,21,24,25]. Considerable amounts of research on key sensing materials involve fine-tuned metal oxides, carbon nanotubes, graphene, dyes and polymers/polymer composites. Among the above materials, polymer-based efforts for the detection of organic vapors have gained wide popularity [1,2,3,4,5,6,17,18,19,20,24]. The large-area processability, as well as widely tailorable physical and chemical properties of polymeric materials has stimulated their exploitation in practical applications.

**Figure 1:**
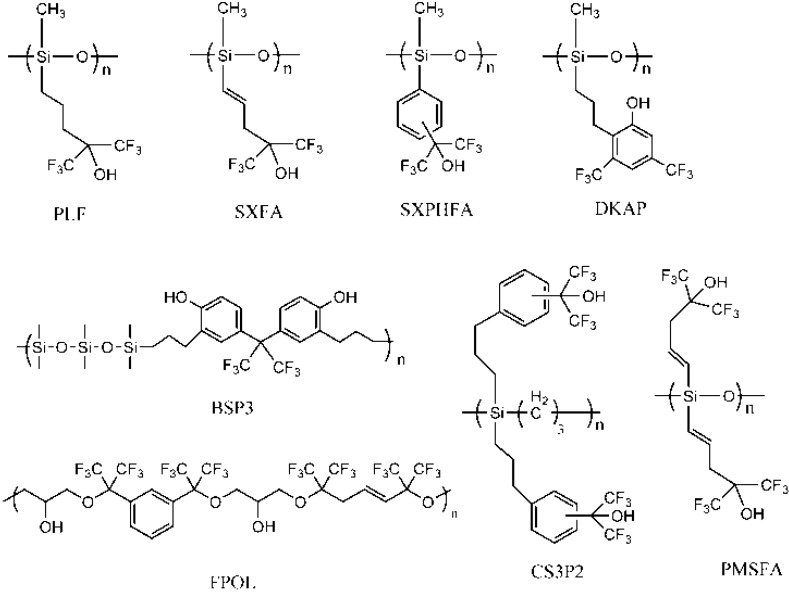
Some typical previously reported HBA polymers.

Strongly hydrogen-bond acidic (HBA) polymers are commonly a set of siloxane- or carbosilane-based molecules functionalized by fluorinated alcohol or fluorinated phenol groups [1,2,3,4,5,6,26,27,28]. Structures of some typical ones are shown in Figure 1. They are essential for the detection of nerve agents originally based on acoustic wave sensors, and have been introduced in microcantilever, chemiresistor and chemicapacitor sensors, and a number of optical approaches [29,30,31,32,33]. In principle, they can interact with the organophosphorus compounds by hydrogen bonding, which promotes the sorption of the analytes into the polymer coating on the transducer surface, and as a consequence, increases the sensor response. Sensitive properties toward nitroaromatic explosives have also been studied with these polymers [17]. The hydrogen bonding is formed by interaction between the nitro group of the explosive compound and the hydroxyl of the functional polymer.

As a representative HBA polymer, linear functionalized polymer (PLF) was introduced by the Demathieu group in 2000, who emphasized the partition coefficients between PLF and a series of reference solutes [34]. Zimmermann initially reported a PLF-coated Love wave device for the detection of organophosphorus compounds, and the sensor showed high sensitivity and reversibility. Subsequently, dimethyl methylphosphonate (DMMP) and sarin (GB) vapor detections were compared to study the different sensitive properties based on an identical Love wave sensor, which demonstrated a higher sensitivity of the sensor to DMMP than to GB [35].

The work detailed here describes our efforts to study the different sorbent properties of a HBA polymer for chemical sensing applications based on a surface acoustic wave (SAW) device. The acoustic sensor works as a frequency-determining electrical oscillator, therefore, the response of the sensor is recognized as the shift of the resonant frequency, which is believed to be primarily attributed to the mass and modulus changes of the sensitive coating [36,37]. The feasibility and high sensitivity of the PLF coated SAW sensor to nitroaromatic explosives had been demonstrated and previously reported by our group [38]. This work focuses on the different sensitive properties of the PLF sensor to real chemical warfare agents and their stimulants, including sarin and its simulant DMMP, mustard gas (HD) and its simulant 2-chloroethyl ethyl sulphide (2-CEES) and 1,5-dichloropentane (DCP). Thereinto, the HBA polymer shows a high sensitivity to 2-CEES, while low sensitivities to HD itself and another simulant DCP. These results are firstly reported and compared here. The real-time responses of the sensor are presented and the corresponding sensitive mechanisms are discussed.

## 2. Experimental Section

Exposure to sarin results in high morbidity and psychological impact, but low mortality. The lethal dose of sarin causing death in 50% of persons exposed (LD_50_) is estimated to be 100 mg·min/m^3^ [39], that is 0.1 mg·min/L, so special safety protocols need to be implemented for exposure to this substance. The synthesis and characterization details of PLF were described in the previous paper [38]. The fabrication of the PLF-coated SAW sensor was performed as follows: A 434-MHz two-port SAW resonator device from Luguang Electronics (Shenzhen, China), was used. PLF was dissolved in chloroform (J & K Scientific, Beijing, China) and spray-coated onto the surface of a bare device by an airbrush apparatus. After film deposition, a frequency shift of 777 kHz was recorded, corresponding to a film thickness of about 27.9 nm. The coated SAW device had a Q value of 3000 and an insertion loss of −14 dB, monitored by a network analyzer (E5070B, Agilent Technologies, Shanghai, China). The coated device, as well as a bare one, were mounted onto a printed circuit board and independently excited in an oscillator loop. The frequency differences between the two devices were obtained by a mixer, and recorded by a SS7200A general counter (Suin Digital Instruments, Shijiazhuang, China).

The test vapors were generated by a MF-3C dynamic vapor generator (China National Metrology Technology Development Co., Beijing, China). The vapors were injected into a small test chamber, where the sensor and a bare device were located in a queue configuration. The chamber was made of aluminium alloy with gas flowing closely over the sensor printed circuit board. The tests were carried out at room temperature (25 °C), and the flow rates of all test vapors were consistently regulated at 1 L/min. The sensitive properties of the sensor were investigated by exposure to various analytes in increasing concentration steps.

## 3. Results and Discussion

The signals were effectively recorded by measuring the change of the shifts of frequency. One test cycle was about 6 min, corresponding to 3-min exposure time to the target vapors and 3-min to pure nitrogen.

### 3.1. Responses to DMMP and Sarin

**Figure 2:**
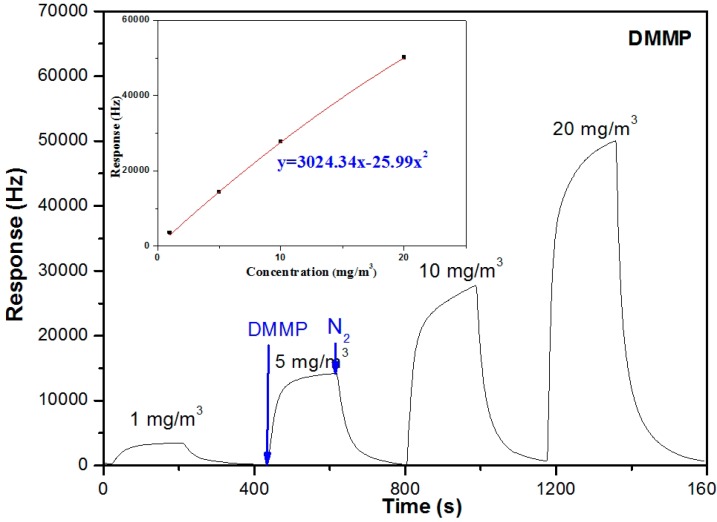
Real-time responses of the PLF sensor to DMMP at concentrations ranging from 1 to 20 mg/m^3^ (3 min DMMP and 3 min N_2_).

We first set out to investigate the responses of the sensor to DMMP and sarin. Figure 2 shows typical responses of the sensor to DMMP at concentrations ranging from 1 to 20 mg/m^3^. As we can see, the response changed immediately when the chamber was purged with DMMP vapor, and positive values were obtained at any test concentration. At the lowest test concentration of 1 mg/m^3^, the response reached 80% maximum of the corresponding peak height within 50 s, and finally achieved a value of 3.1 kHz. When pure nitrogen was injected into the chamber, desorption took place rapidly as well and the response could return to the original value. The signals recorded in the curve showed that each adsorption–desorption test cycle exhibited an analogous square wave shape.

The inset of Figure 2 shows the response of the sensor as a function of the vapor concentration. PLF is a viscous liquid at room temperature and the responses will be influenced by the viscoelastic effect of the polymer. Thus a parabolic fitting was chosen instead of the linear fit, which is more suitable for pure gravimetric devices like the quartz crystal microbalance (QCM). After polynomial fitting, an expression of Δ*f* = *3024.34x* − *25.99x^2^* is obtained where Δ*f* is in Hz and vapor concentration *x* is in mg/m^3^. According to this expression, the response of the sensor at 0.009 mg/m^3^ is estimated to be 28 Hz. Thus, the limit of detection (LOD) of the sensor, taking into account the signal-to-noise ratio of 3:1, is deducted to be 0.009 mg/m^3^ since the noise level of the coated SAW sensor is around 5–9 Hz.

**Figure 3:**
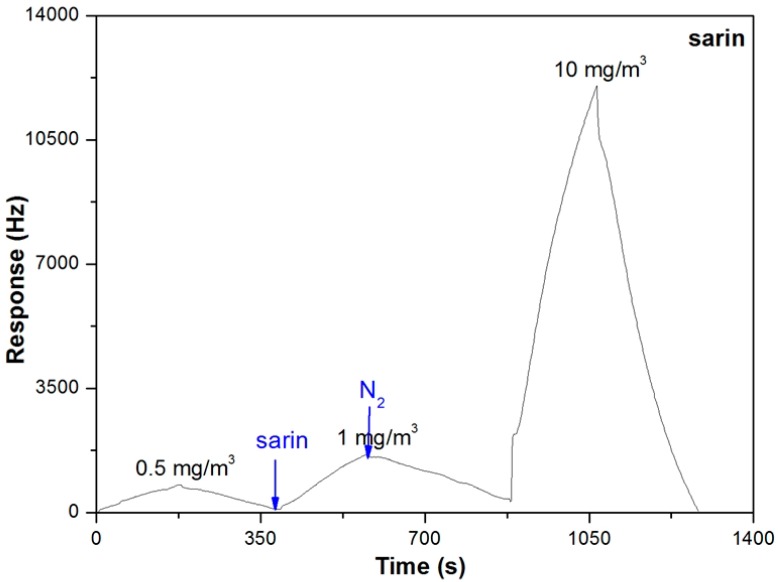
Real-time responses of the sensor to sarin at the concentrations of 0.5, 1 and 10 mg/m^3^ (3 min sarin and 3 min N_2_).

**Figure 4:**
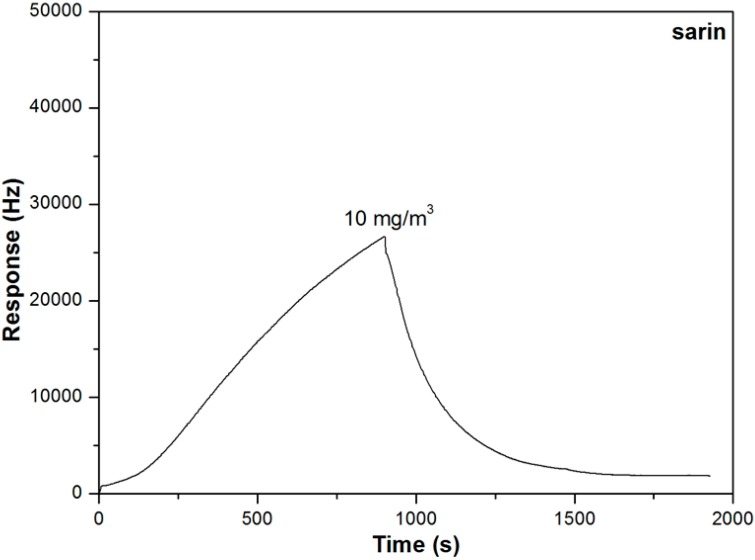
Response of the sensor to sarin at the concentration of 10 mg/m^3^ (16 min sarin and 16 min N_2_).

Figure 3 shows the responses of the sensor to sarin at the concentrations of 0.5, 1 and 10 mg/m^3^. Unlike DMMP, the responses of the sensor to sarin exhibited a triangular-wave curve for each sorption–desorption cycle. The response value increased linearly with the exposure time to the sarin vapor with a time-dependent sensitivity of 260 Hz/min at the concentration of 0.5 mg/m^3^, which indicated that PLF coating couldn’t reach an equilibrium state within an exposure of 3 min. Actually, at the highest test concentration of 10 mg/m^3^, the response value of the sensor could reach 26 kHz at 16 min, which wasn’t even a steady state, shown in Figure 4.

The response comparison of a poly(methyl-{3-[2-hydroxy-4,6-2(3methyl)]phenyl}-propyl siloxane (DKAP)-coated SAW sensor to DMMP and sarin has already been investigated by our group [2]. Like PLF, the response of the DKAP sensor to sarin is slower than to DMMP. The reason leading to the result is the size discrepancy of the two analytes and the hydrogen bond strength.

### 3.2. Responses to 2-CEES, DCP and HD

**Figure 5:**
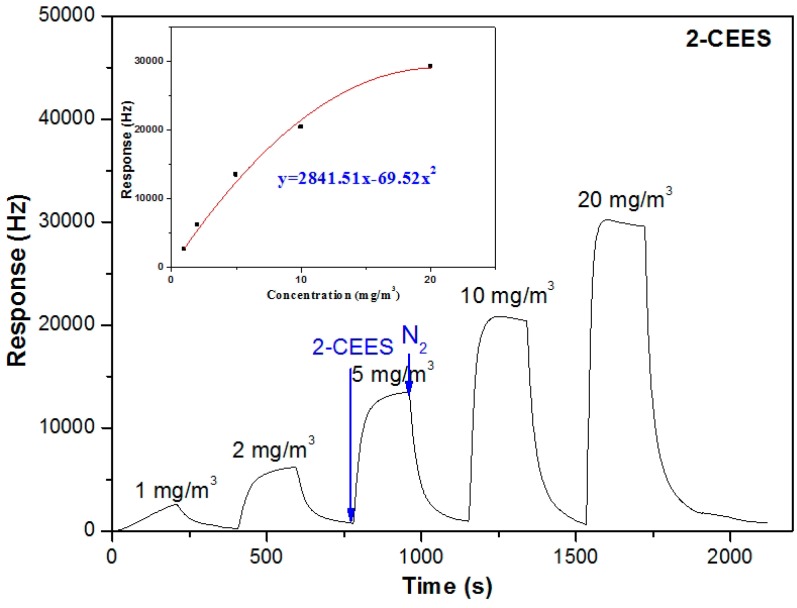
Response variations of the PLF sensor in terms of the concentration change of 2-CEES (3 min 2-CEES and 3 min N_2_).

Figure 5 includes the response variations in terms of the concentrations of 2-CEES. Like the DMMP tests, at all test concentrations, the sensor shows fast adsorption processes during the exposure to 2-CEES vapor. Thereinto, the responses of the sensor towards 2-CEES vapor at the concentrations of 1, 2 and 5 mg/m^3^, could reach steady states which represent the adsorption-desorption equilibrium of the sensitive coating. Mass effects play a primary role in the frequency shift response under such conditions. However, an interesting phenomenon is onserved in that the responses decline slightly after reaching maximum values at relatively high test concentrations of 10 and 19 mg/m^3^. These adsorption kinetic behaviors are interpreted in terms of the viscoelastic effect, which is based on the swelling-induced modulus changes of the PLF coating [36,37]. Thus, in a sense, the modulus changes could offset the response values of the sensor induced by the mass effect. These results indicate that both the mass effect and viscoelastic effect contribute to the frequency shift response when exposed to relatively high concentrations of 2-CEES. The inset of Figure 5 also shows the response of the sensor as a function of the vapor concentration. An expression of Δ*f = 2841.51x − 69.52x^2^* is obtained. According to this expression, the response of the sensor at 0.01 mg/m^3^ is estimated to be 28 Hz. Thus, the LOD of the sensor to 2-CEES is deducted to be 0.01 mg/m^3^.

**Figure 6:**
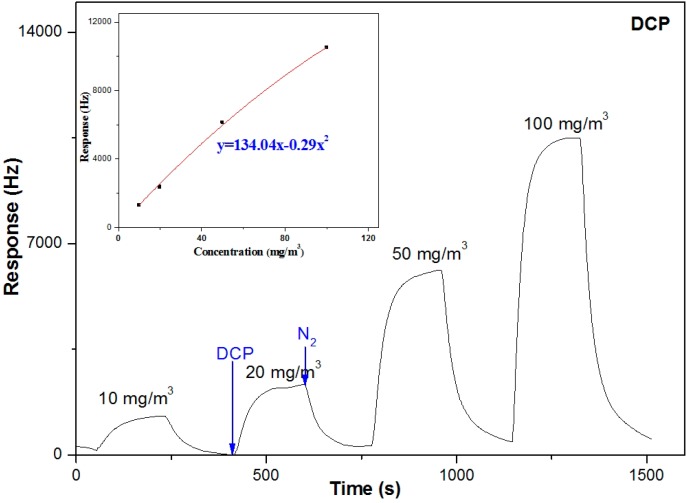
Real-time responses of the PLF sensor to DCP (3 min DCP and 3 min N_2_).

Two of the common simulants for HD, 2-CEES and 1,5-dichloropentane (DCP) have been studied with various sensitive materials [40,41,42,43]. However, to the best of our knowledge, none of these studies concerned the discrepancy between the sensitive properties of the two compounds. Our work indicated that the PLF sensor showed a nearly 20 times higher response to 2-CEES than to DCP at the test concentration of 10 mg/m^3^, as shown in Figure 6. The inset of Figure 6 shows the response of the sensor as a function of the vapor concentration. An expression of Δ*f = 134.04x − 0.29x^2^* is obtained. According to this expression, the response of the sensor at 0.21 mg/m^3^ is estimated to be 28 Hz. Thus, the LOD of the sensor to DCP is deducted to be 0.21 mg/m^3^.

**Figure 7:**
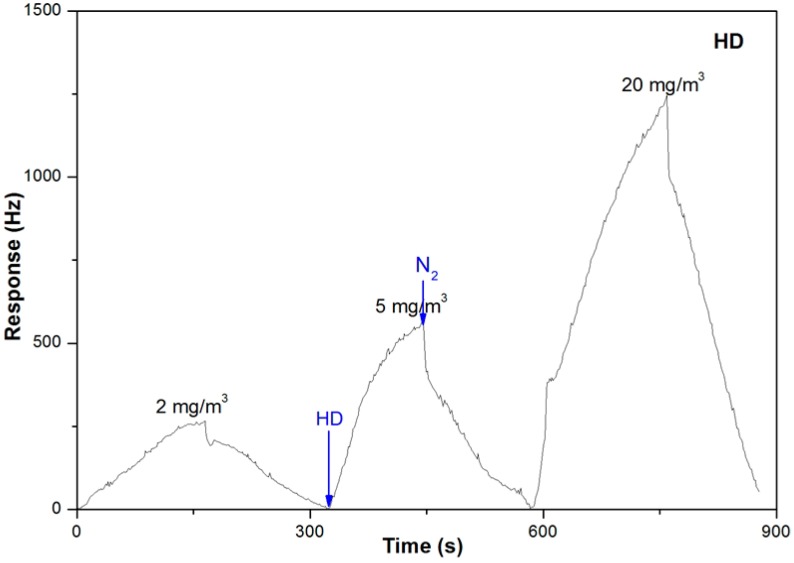
Responses of the PLF sensor to HD at the concentrations of 2, 5 and 20 mg/m^3^ (3 min HD and 3 min N_2_).

A further study on the sensitive property of the sensor to HD has also been conducted. Figure 7 is the plot for the response of the sensor with the variation of HD concentration. Like DCP, the sensor showed a low sensitivity of about 500 Hz to HD at the test concentration of 5 mg/m^3^, which was only 1/20 as compared to 2-CEES. Furthermore, the response rate of the sensor to HD was much slower than to 2-CEES.

**Figure 8:**
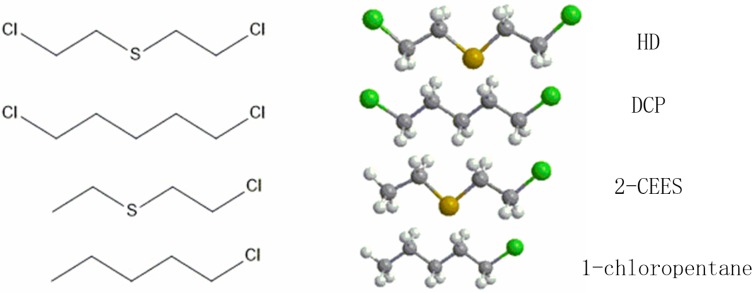
Molecular structures of HD, its simulants and 1-chloropentane.

The molecular structures of the three compounds are shown in Figure 8. Compared to HD, a chlorine atom and a sulphur atom are lacking in the molecular structures of 2-CEES and DCP, respectively. The chlorine atom shows strong electronegativity and the sulphur atom is electron-rich. The participation of the two atoms results in different polarities and electron cloud distributions of the three molecules, which may explain the discrepancy in the sensitive behavior of the sensor.

In a word, the results indicate that the PLF sensor shows tremendously different sensitive behaviors to chemical warfare agents and their simulants, which means that the sensitivity studies of the sensors to the simulants of the real agents may not be suitable for practical application.

### 3.3. Responses to Interfering Vapors

**Figure 9:**
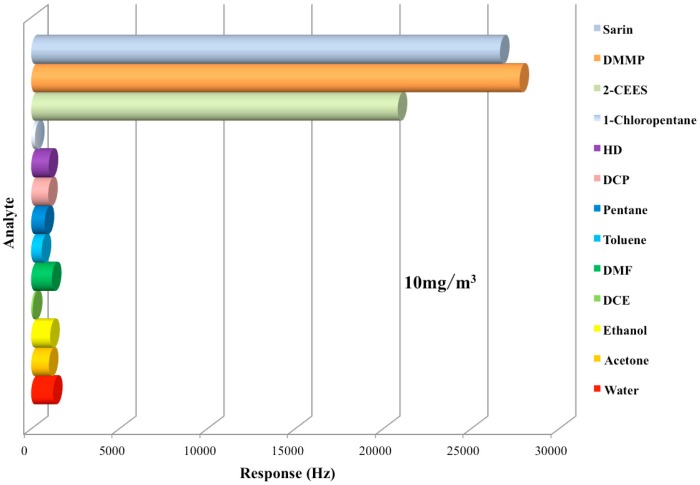
Responses of the PLF sensor to various analytes at 10 mg/m^3^.

Responses of the sensor to various vapors are shown in Figure 9 and Table 1. Thereinto, DMMP, sarin and 2-CEES are organic vapors that possess flexible electron-rich cloud around oxygen or sulphur atom. The rest interference vapors can be fallen into different categories by the functional groups in their molecular structures. Ethanol and acetone are oxidized polar hydrocarbon, pentane and toluene are low polar hydrocarbons, 1,2-dichloroethane (DCE) is halogenated non-polar hydrocarbon, 1-chloropentane is halogenated polar hydrocarbon and N,N-dimethylformamide (DMF) is nitric polar hydrocarbon.

**Table 1:** Sensor responses to various vapors at 10 mg/m^3^.

		**Acetone**	**Ethanol**	**DCE**	**DMF**	**Toluene**	**Pentane**	**DCP**
Response (Hz)	test 1	998	1059	99	1165	582	767	951
	test 2	1011	1089	103	1184	599	759	975
	test 3	1006	1081	98	1178	596	765	969
Pressure at 25 ℃ (mmHg)		224.8	58.4	99.9	5.5	31.5	512.8	1.1
		**HD**	**1-chloro-pentane**	**2-CEES**	**DMMP**	**Sarin**		
Response (Hz)	test 1	996	209	20754	27642	26601		
	test 2	991	213	20791	27706	26655		
	test 3	997	217	20837	27761	26651		
Pressure at 25 ℃ (mmHg)		0.1	30.04	3.4	0.833	0.4		

The response values were obtained at a consistent test concentration of 10 mg/m^3^. As we can see, responses of the sensor to the three electron-rich compounds were obviously larger than to the interferent vapors. The response to water vapor was also investigated in this paper and former work [44]. Obviously, compared to the non-polar and low polar compounds, the sensor was more sensitive to the polar analytes.

### 3.4. Sensitive Mechanism

**Figure 10:**
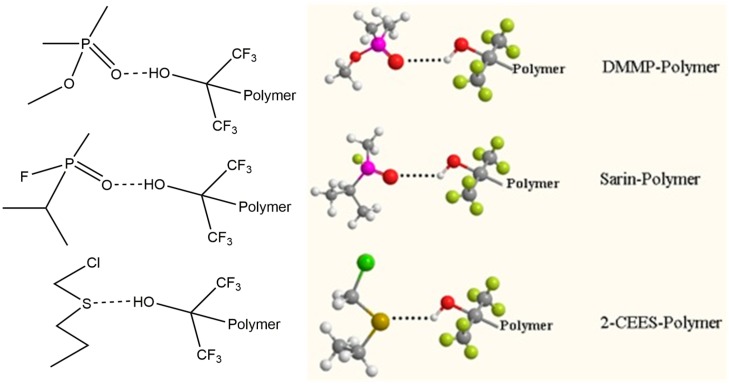
Hydrogen-bonding interactions between DMMP, sarin, 2-CEES and HBA polymer PLF.

It is well established that the interactions between HBA polymers and organophosphorus are based on hydrogen bonding, as shown in Figure 10 [3,4,5].

For the pendant group of PLF, the electron withdrawing effect of the two adjacent trifluoromethyl groups increases the activity of the hydroxyl group, which greatly promotes the hydrogen bond acidity of the HFIP group. As to DMMP and sarin, the oxygen atom shows a higher electonegativity than the phosphorus atom, thus, the strong electron-withdrawing properties lead to a high density of the electron cloud of the oxygen atom. When contacting the HBA polymer, the electron-rich oxygen atom can act as an electron donor. As a result, hydrogen bonding is formed between the pendant group of PLF and the oxygen atoms of DMMP or sarin.

As shown in Figure 9, 2-CEES showed a response of 20.8 kHz at 10 mg/m^3^, which is much larger than its similar counterparts HD (0.99 kHz) and DCP (0.97 kHz). The saturation vapor pressures of 2-CEES, HD and DCP are 3.4, 0.1059 and 1.125 Torr, respectively. As for pure physical adsorption, the lower the saturation vapor pressure, the easier the vapor swelling into the adsorption material [45]. Obviously the adsorption of 2-CEES is far more than physical adsorption. The reason is speculated to be that the lone pair electrons on the sulphur atom can form weak hydrogen bonding with the hydroxyl in PLF. As is known, the ethyl and the chlorethylidene groups are electron-donating and electron-withdrawing groups, respectively. For HD, the electron-withdrawing effect of the two symmetric chlorethylidene groups decreases the density of electron cloud on the sulphur atom collaboratively, which is believed to lead to the low sensitivity of the sensor.

The mechanism is confirmed by a comparison test to 1-chloropentane. The only difference between 1-chloropentane and 2-CEES is the sulphur atom in 2-CEES while a carbon atom occupies that position in 1-chloropentane. Since the PLF sensor shows a much higher sensitivity to 2-CEES than to 1-chloropentane, as shown in Figure 9, we can safely draw a conclusion that the sulphur atom plays a vital role in the 2-CEES adsorption, by providing the hydrogen-bonding site. Furthermore, Hussain *et al.* [46] also found that the surface hydroxyl groups of Ag/TiO_x_–Al_2_O_3_ absorb sulfur aromatics primarily via hydrogen-bonding, which is consistent with our findings.

Another reason might be that the active hydroxyl group in PLF can form ion-dipole interactions with 2-CEES, thus the 2-CEES molecule shows remarkable polarity arising from the asymmetry of the molecular structure, while as to HD and DCP, their polarity is very tiny. However, as shown in Figure 9, the sensor exhibited low sensitivity to ethanol, DMF and 1-chloropentane, which are all polar compounds. Thus, the dipole-dipole interaction is a synergic factor instead of a primary factor that explains to the high responses of the sensor when exposed to DMMP, sarin and 2-CEES.

## 4. Conclusions

A HBA polymer (PLF)-coated SAW sensor was introduced for the detection a variety of analytes, including DMMP, sarin and 2-CEES. Care was taken to investigate the sensitive properties of the sensor to these analytes. High sensitivity of the sensor is demonstrated and the reason is interpreted as the formation of hydrogen bonding between PLF and the analytes that have electron-rich atoms. Each of these compounds possesses an electron-rich site that acts as an electron donor, which can interact with the hydroxyl group of PLF by hydrogen bonding. However, the sensor shows tremendously different sensitive behaviors to real agents and their simulants, which is believed to due to the discrepancy in their molecular structures. The results show that the sensitivity study of the sensor to the simulants of the real agents is not enough for practical application. Furthermore, we will evaluate the feasibility of the sensor for the detection of other organosulphur compounds, such as dimethyl sulphide and methyl disulfide, in continuing efforts.
